# An Open‐Label Study to Evaluate the Effect of Eluxadoline on the Single‐Dose Pharmacokinetics of Midazolam in Healthy Participants

**DOI:** 10.1002/cpdd.1150

**Published:** 2022-08-08

**Authors:** Ramesh Boinpally, Danielle McGeeney, Edward Kaczynski, Darren Weissman

**Affiliations:** ^1^ AbbVie Inc Madison New Jersey USA; ^2^ AbbVie Inc North Chicago Illinois USA

**Keywords:** diarrhea, drug‐drug interaction, eluxadoline, irritable bowel syndrome, midazolam

## Abstract

Eluxadoline is a mixed μ‐opioid, κ‐opioid receptor agonist, and δ‐opioid receptor antagonist, approved in the United States for adults with diarrhea‐predominant irritable bowel syndrome. This phase 1, single‐center, open‐label, single‐sequence study was conducted on 30 healthy participants to establish whether steady‐state eluxadoline increases systemic exposure of the cytochrome P450 (CYP) 3A4 substrate midazolam. Participants received oral midazolam 4 mg on day 1 with a 7‐day washout period. On days 8‐16, oral eluxadoline 100 mg was administered twice daily. On day 15, midazolam 4 mg was coadministered with the eluxadoline 100‐mg morning dose. Primary outcome measures were pharmacokinetic parameters of midazolam and 1‐hydroxy‐midazolam. The midazolam and 1‐hydroxy‐midazolam geometric mean ratios and 90%CIs for maximum plasma drug concentration were 99.0% (91.6‐107.0) and 113.8% (104.9‐123.5), respectively, and area under the plasma concentration–time curves were 90.5% (83.9‐97.6) and 105.1% (99.8‐110.7), respectively, demonstrating the 2 treatments were bioequivalent, and there was no clinically significant drug interaction. All treatment‐emergent adverse events were treatment related, mild in intensity, with no serious adverse events. These results suggest that eluxadoline has no clinically significant effect on CYP3A4 activity and is, therefore, unlikely to affect the pharmacokinetics of other CYP3A4 substrates.

Eluxadoline is a mixed μ‐opioid and κ‐opioid receptor agonist and δ‐opioid receptor antagonist that has local gastrointestinal (GI) activity and low oral bioavailability.^1^, ^2^, ^3^, ^4^ The beneficial effects of eluxadoline in treating diarrhea‐predominant irritable bowel syndrome (IBS‐D) derive from its local action within the GI tract, where the extensive expression of opioid receptors plays a key role in regulating GI motility, secretion, and visceral sensation.^1^, ^2^, ^5^ The mixed opioid pharmacology of eluxadoline appears to allow it to effectively improve abdominal pain and stool consistency in patients with IBS‐D while mitigating the risk of constipation.

The effectiveness of eluxadoline to treat the symptoms of IBS‐D was demonstrated in 2 large, randomized, phase 3 clinical studies.^6^, ^7^, ^8^ The RELIEF phase 4 study confirmed the efficacy of eluxadoline in a population with IBS‐D who reported previous inadequate response to loperamide.^9^ The study showed that a significantly greater proportion of patients treated with eluxadoline achieved the composite responder end point compared with placebo (22.7% vs 10.3%, respectively), which was defined as an improvement of ≥40% in worst abdominal pain in the preceding 24 hours, and a Bristol Stool Form Scale score of <5 for ≥50% of treatment days. Eluxadoline is approved in the United States for the treatment of adults with IBS‐D, which is a subtype of irritable bowel syndrome.^1^

Eluxadoline is minimally absorbed following oral administration, with peak plasma concentrations (C_max_) reached at a median time of maximum plasma drug concentration (t_max_) of 1.5 hours.^10^ Due to its low oral bioavailability, the C_max_ of eluxadoline is ≈2‐4 ng/mL, and the area under the plasma concentration–time curve (AUC) is 12‐22 ng • h/mL after a 100‐mg dose.^10^, ^11^, ^12^ Eluxadoline displays dose‐linear pharmacokinetics (PK) with no accumulation upon repeated twice‐daily dosing. This lack of accumulation is consistent with the short terminal elimination half‐life (≈5 hours).^10^, ^11^

A phase 1 study found higher systemic exposure of eluxadoline (single dose, 100 mg) in volunteers with hepatic impairment compared with healthy volunteers.^12^ Eluxadoline is contraindicated for patients with severe hepatic impairment, and a lower dose (75 mg) is recommended for individuals with mild to moderate hepatic impairment.^1^ Additionally, another study found that eluxadoline did not cause QT interval prolongation in healthy male and female volunteers at therapeutic (100‐mg) and supratherapeutic (1000‐mg) doses.^13^

In human and nonrodent cell lines, the predominant in vitro metabolic pathway is direct glucuronidation of the methoxy‐benzoic acid moiety to form the acyl glucuronide M11. M11 is also the major metabolite identified in human intestinal microsomal incubations. In vivo metabolism studies found the majority of eluxadoline is excreted in the feces of animals unchanged, while the major metabolite, M11, is seen in the urine of rats, primates, and humans. M11 was also detected in plasma samples from monkeys (data not published).

In vitro studies indicate that eluxadoline is neither an inducer of cytochrome P450 (CYP) 1A2, CYP2C9, CYP2C19, or CYP3A4, nor an inhibitor of CYP1A2, CYP2A6, CYP2B6, CYP2C9, CYP2C19, or CYP2D6 at clinically relevant systemic concentrations. Although CYP2E1 was slightly inhibited by eluxadoline (half maximal inhibitory concentration, ≈20 micromolar [11 μg/mL]), clinically meaningful interactions are unlikely. In an additional in vitro study using human liver and intestinal microsomes investigating the potential of eluxadoline to inhibit the metabolism of concomitantly administered drugs, eluxadoline demonstrated a metabolism‐dependent (ie, nicotinamide adenine dinucleotide phosphate–dependent) inhibition of CYP3A4/5 activity (Allergan Report Number ELX‐PH‐04; data not published).

Eluxadoline was shown to be transported by organic anion transporter (OAT) 3, OATP1B1, and bile salt export pump at 400 ng/mL, which is 133 times the C_max_ of the 100‐mg therapeutic dose, and by multidrug resistance protein‐2 at all tested concentrations (4‐400 ng/mL).^11^ Eluxadoline (400 ng/mL) did not inhibit any drug transporters tested, with the exception of OATP1B1. Coadministration of cyclosporine increased plasma levels of eluxadoline, supporting eluxadoline as an in vivo substrate of OATP1B1, which plays a major role in the oral absorption (hepatic first‐pass extraction) and excretion (biliary clearance) of eluxadoline.^11^ A drug‐drug interaction (DDI) study with rosuvastatin, an OATP1B1 substrate, showed that there was no clinically relevant interaction, as there was only an ≈1.4‐fold increase in rosuvastatin AUC (data not published).

Midazolam is a short‐acting imidazobenzodiazepine, which is extensively metabolized by CYP3A in the liver and intestine.^14^ As midazolam is not a substrate of P‐glycoprotein, it is a sensitive CYP3A probe drug for evaluating the effect of an inhibitor or inducer on CYP3A activity in vivo.^15^ This study was conducted to establish whether eluxadoline, at its highest clinical dose of 100 mg twice daily, increases the systemic exposure of the concomitantly administered CYP3A4 substrate midazolam after 1 week in healthy participants.

## Methods

### Patient Population and Study Design

This was a phase 1, single‐center, open‐label, single‐sequence study in healthy participants, conducted from November 2016 to January 2017. The main inclusion criteria were healthy men and women aged 18‐45 years. The full inclusion and exclusion criteria are listed in Table S1.

**Figure 1:**
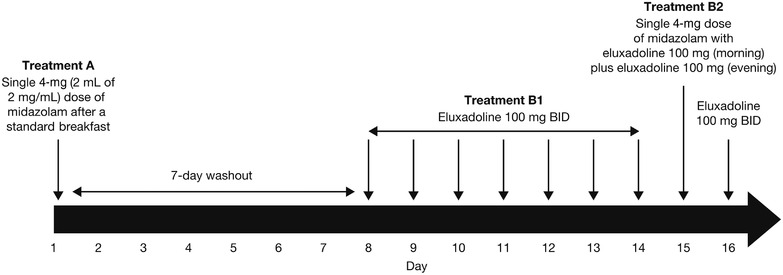
Study design. BID, twice daily.

Participants enrolled in the study were assigned to a single, fixed‐treatment sequence of a single 4‐mg (2 mL of 2 mg/mL oral syrup [Roxane Laboratories, Inc., Columbus, Ohio]) dose of midazolam on day 1 (treatment A), which was administered with 240 mL of water, starting at ≈8:00 am on day 1 following a standard breakfast given at 7:30 am. This was followed by a 7‐day washout period (Figure 1). On days 8‐14, participants received an oral tablet of eluxadoline 100 mg twice daily (treatment B1). On day 15, a single 4‐mg (2 mL of 2 mg/mL oral syrup) dose of midazolam was coadministered with eluxadoline 100 mg in the morning and then eluxadoline 100 mg was given in the evening, followed by eluxadoline 100 mg twice daily on day 16 (treatment B2). Eluxadoline was administered with 240 mL of water, starting at ≈8:00 am and 8:00 pm on days 8‐16 following a standard meal given at 7:30 am and 7:30 pm, respectively.

This study was conducted in accordance with the International Council for Harmonisation E6 guideline for good clinical practice and the principles of the Declaration of Helsinki. The institutional review board (IntegReview IRB) approved the study protocol for the site (Clinical Pharmacology of Miami, LLC, Hialeah, Florida), and written informed consent was obtained from all participants at the first study visit.

### Rationale of Dose Selection

The dose of eluxadoline 100 mg twice daily on days 8‐16 was selected to ensure the maximum possible CYP3A4 inhibition by eluxadoline. Comparison of midazolam PK on day 1 vs day 15 helped to estimate the CYP3A4 inhibition potential of eluxadoline. A single 4‐mg dose of midazolam was chosen for the study based on a previous food‐effect study that tested a 15‐mg oral dose, and suggested that a 4‐mg dose would have an adequate safety margin in the event of increased midazolam exposure due to potential CYP3A4 inhibition by eluxadoline.^16^

### Study Objectives and End Points

The primary objective of the study was to evaluate the effect of eluxadoline on the single‐dose PK of midazolam in healthy participants. The secondary objectives were to evaluate the safety and tolerability of single‐dose midazolam when administered alone and in combination with eluxadoline.

Primary outcome measures were PK parameters of midazolam and 1‐hydroxy‐midazolam (α‐hydroxy‐midazolam) metabolite derived from plasma concentrations following treatments A (day 1) and B2 (day 15) (see Supplemental Information, Measurements for further details). Specific parameters evaluated were AUC from time 0 to time t and AUC from time 0 to infinity, C_max_, t_max_, terminal elimination rate constant, terminal elimination half‐life, and apparent total body clearance of drug from plasma after extravascular administration (for midazolam only). In addition, the ratio of 1‐hydroxy‐midazolam to midazolam for C_max_ and AUC were calculated following treatments A and B2. Sampling for eluxadoline plasma concentration was performed before the morning doses on days 8, 13, 14, and 15 (see Supplemental Information, Measurements for further details).

Safety measures included adverse events (AEs), clinical laboratory determinations, vital sign parameters, electrocardiogram (ECG) results, and physical examination findings (see Supplemental Information, Measurements for further details).

### Statistical Analyses

#### Determination of Sample Size

A sample size of 24 participants was determined to provide 95% power to show that the 90%CIs for the point estimate of the ratio of geometric mean PK parameters (C_max_ and AUC) of midazolam with and without coadministration of eluxadoline are within 80%‐125%. This was based on the assumption of a within‐subject coefficient of variation of 22% for C_max_ and AUC of midazolam, and a true ratio of test to reference geometric means of ≈1.07. A total of 30 participants were enrolled to account for possible dropouts.

#### Analysis Populations

Two populations were considered for the statistical analysis of this study. The safety population consisted of all participants who received at least 1 dose of study treatment. The PK population consisted of all participants who had evaluable PK parameters of midazolam.

#### Pharmacokinetic Analyses

The principal parameters describing the PK of midazolam and 1‐hydroxy‐midazolam metabolite were derived from plasma concentrations using noncompartmental analysis with the software program Phoenix WinNonlin version 6.2 (Certara, Princeton, New Jersey). Plasma concentrations below the limit of quantification were treated as 0 for all PK calculations. The actual sampling times were used in the calculations of PK parameters in this study (see Supplemental Information, Measurements for further details).

Descriptive statistics were provided by treatment for plasma concentrations of midazolam and 1‐hydroxy‐midazolam metabolite for participants in the PK population. Descriptive statistics were also provided for plasma eluxadoline concentrations.

PK parameters (C_max_, AUC from time 0 to time t, and AUC from time 0 to infinity) of midazolam and 1‐hydroxy‐midazolam metabolite were compared using a linear mixed‐effects model with treatment as a fixed effect and participant as a random effect. The 2‐sided 90%CI for the ratio of AUC and C_max_ geometric means between treatments were constructed. The difference in median t_max_ between midazolam plus eluxadoline vs midazolam alone was determined.

#### Safety Analyses

For each safety parameter, the last assessment made before the first dose of study treatment was used as the baseline for all end‐of‐study analyses. The last assessment made before the first dose of study treatment in each treatment period was used as the baseline for AE assessments during PK profiling periods.

## Results

### Patient Disposition and Demographics

Thirty participants were enrolled in the study and received midazolam on day 1; 29 participants received eluxadoline on days 8‐14, and received eluxadoline and midazolam on day 15 and eluxadoline on day 16. A total of 29 participants completed all the planned treatments and were included in the PK population, with 1 participant lost to follow‐up after day 3.

Of the 30 participants in the safety population, the mean age was 31.8 years (Table 1). The majority of participants were men (n = 18) and Hispanic or Latino (n = 18), with a mean baseline body mass index of 25.9 kg/m^2^.

**Table 1:** Demographics and Baseline Characteristics

	All Participants (N = 30)
Age, y, mean (SD)	31.8 (8.3)
Sex, male, n (%)	18 (60.0)
Race/ethnicity, n (%)	
White	17 (56.7)
Black or African American	13 (43.3)
Hispanic or Latino	18 (60.0)
Weight, kg, mean (SD)	73.9 (12.0)
Height, cm, mean (SD)	168.5 (8.1)
Body mass index, kg/m^2^, mean (SD)	25.9 (3.2)

SD, standard deviation.

### Pharmacokinetics

#### Midazolam and 1‐Hydroxy‐Midazolam

**Figure 2:**
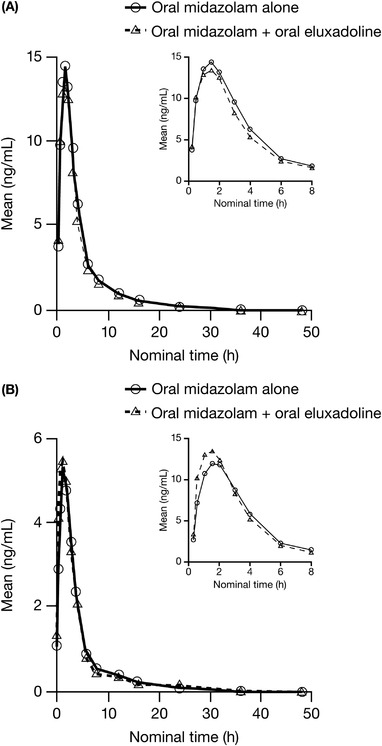
Mean plasma concentrations of (A) midazolam and (B) 1‐hydroxy‐midazolam after oral administration of 4 mg of midazolam, either alone or in the presence of steady‐state eluxadoline.

The mean peak plasma concentrations of midazolam were similar in the absence or presence of eluxadoline (Table 2 and Figure 2). The mean peak plasma concentration of 1‐hydroxy‐midazolam was ≈14% higher following midazolam administration in the presence of eluxadoline.

**Table 2:** Arithmetic Mean (SD) Pharmacokinetic Parameters of Midazolam and 1‐Hydroxy‐Midazolam Following a Single Dose of Midazolam 4 mg Either Alone or in the Presence of Steady‐State Eluxadoline in Healthy Participants

Parameter	Midazolam Alone (Treatment A) N = 29	Midazolam + Eluxadoline (Treatment B2) N = 29	Ratio of Geometric Means, %	90%CI
Midazolam				
C_max_, ng/mL	15.7 (6.2)	15.6 (6.0)	99.0	91.6‐107.0
AUC_0‐t_, ng • h/mL	68.4 (40.6)	62.7 (40.9)	90.5	83.9‐97.6
AUC_0‐∞_, ng • h/mL	69.5 (41.7)	63.8 (42.2)	90.3	83.9‐97.3
t_max_,^a^ h	1.5 (0.5‐3.0)	1.5 (0.3‐2.0)	…	…
t_½_, h	6.0 (1.5)	5.6 (1.8)	…	…
CL/F, L/h	69.7 (28.0)	77.9 (32.5)	…	…
1‐hydroxy‐midazolam				
C_max_, ng/mL	5.5 (3.9)	6.3 (4.1)	113.8	104.9‐123.5
AUC_0‐t_, ng • h/mL	23.4 (21.9)	24.0 (20.1)	105.1	99.8‐110.7
AUC_0–∞_, ng • h/mL	24.1 (22.4)	25.3 (20.4)	107.0	101.8‐112.5
t_max_,^a^ h	1.56 (0.5‐3.0)	1.5 (0.5‐2.0)	0	…
t_½_, h	6.0 (1.9)	8.6 (4.7)	…	…

AUC_0‐∞_, area under the plasma concentration–time curve from time 0 to infinity; AUC_0‐t_, area under the plasma concentration–time curve from time 0 to time t; CL/F, apparent total body clearance of drug from plasma after extravascular administration; C_max_, maximum plasma drug concentration; SD, standard deviation; t_½_, terminal elimination half‐life; t_max_, time of maximum plasma drug concentration.

Treatment A: single‐dose midazolam 4 mg alone on day 1; treatment B1: eluxadoline 100 mg twice daily on days 8‐14; treatment B2: single‐dose midazolam 4 mg with eluxadoline 100 mg twice daily on days 15 and 16.

^a^ Median (min‐max).

The AUC of midazolam was ≈10% lower following its administration with eluxadoline. The mean AUC of 1‐hydroxy‐midazolam was ≈7% higher following midazolam administration with eluxadoline.

The 90%CIs for the geometric mean ratios of C_max_ and AUC parameters of midazolam and 1‐hydroxy‐midazolam demonstrated that the 2 treatments were bioequivalent, and there was no clinically significant drug interaction.

#### Eluxadoline

**Figure 3:**
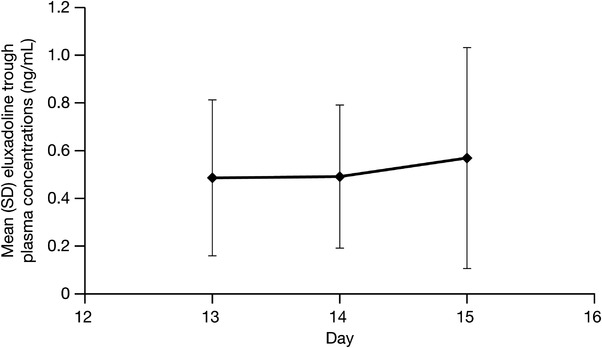
Mean (SD) trough plasma eluxadoline concentrations (ng/mL) following oral eluxadoline 100 mg twice‐daily administration on days 8‐14 in healthy participants. SD, standard deviation.

Steady‐state plasma concentrations of eluxadoline 100 mg twice daily in healthy participants were achieved after 5 days of twice‐daily dosing (Figure 3).

#### 1‐Hydroxy‐Midazolam to Midazolam Ratio

The mean 1‐hydroxy‐midazolam to midazolam ratios were slightly higher following midazolam administration in the presence of eluxadoline compared with midazolam alone, but the difference was unlikely to be clinically significant.

### Safety

A total of 12 participants (40.0%) experienced treatment‐emergent AEs (TEAEs) following administration of treatment A; 9 participants (31.0%) experienced TEAEs following treatment B1; and 8 participants (27.6%) experienced TEAEs following treatment B2 (Table 3). All TEAEs were assessed by the investigator as treatment related and were mild in intensity. The most common TEAE was hypersomnia (treatment A, 36.7% [n = 11]; treatment B2, 27.6% [n  =  8]). There were no serious AEs, and no TEAEs led to discontinuation of study treatment. There were no deaths reported during the study.

**Table 3:** Treatment‐Emergent Adverse Events (Safety Population)

n (%)	Midazolam Alone (Treatment A), N = 30	Eluxadoline Alone (Treatment B1), N = 29	Midazolam + Eluxadoline (Treatment B2), N = 29
All TEAEs	12 (40.0)	9 (31.0)	8 (27.6)
Preferred Term			
Gastrointestinal disorders	0	5 (17.2)	0
Constipation	0	5 (17.2)	0
Nervous system disorders	12 (40.0)	6 (20.7)	8 (27.6)
Hypersomnia	11 (36.7)	0	8 (27.6)
Headache	0	5 (17.2)	1 (3.4)
Somnolence	1 (3.3)	1 (3.4)	0

TEAE, treatment‐emergent adverse event.

Events were coded using the Medical Dictionary for Regulatory Activities version 19.1. Participants are counted only once within each System Organ Class and Preferred Term. All TEAEs were assessed by the investigator as treatment related and were mild in intensity.

There were no clinically significant changes from normal baseline values to the end of the study in serum chemistry parameters, and none were assessed as associated with an AE. Overall, there were few changes to hematology or coagulation parameters during the study. Two participants had potentially clinically significant hematology values at the end of the study. One participant had a low baseline hemoglobin of 114 g/L, which decreased to 107 g/L at the end of the study. The other participant had a normal baseline hematocrit of 0.38 and hemoglobin of 120 g/L, which decreased to low values of 0.31 and 102 g/L, respectively, at the end of the study. This participant also experienced a TEAE of mild hypersomnia with onset and resolution on day 1. There were no potentially clinically significant changes in vital signs or ECG results at the end of the study, and no participants had potentially clinically significant results during the study.

## Discussion

In vitro data had suggested that eluxadoline inhibits CYP3A4.^17^, ^18^ Because eluxadoline may be prescribed in combination with CYP3A4 substrates, it is important to know if such combinations can be administered with or without dose adjustments. This DDI study was conducted with midazolam, a CYP3A4 substrate, to assess the potential CYP3A4 inhibitory effect of eluxadoline in humans and demonstrated that oral administration of eluxadoline at steady state in healthy humans had no significant effect on the PK of orally administered midazolam and its metabolite 1‐hydroxy‐midazolam. Multiple dosing with eluxadoline did not change the C_max_ of midazolam, and AUC decreased by 10%. For 1‐hydroxy‐midazolam, coadministration of eluxadoline increased the C_max_ by 14% and AUC by 7%. None of these changes would be clinically significant.

The results of this study explain why there have not been any clinically relevant DDI observed between eluxadoline and oral contraceptives in healthy volunteers.^1^ It is therefore likely that eluxadoline has no clinically significant effect on CYP3A4 activity, does not affect the PK of other CYP3A4 substrates, and no dose adjustments are needed for CYP3A4 substrates when coadministered with eluxadoline.

Overall, each treatment regimen was well tolerated, with all TEAEs mild in intensity. There were no reports of serious AEs or AEs leading to the discontinuation of eluxadoline, and there were no deaths during the study. In addition, no potentially clinically meaningful changes in serum chemistry parameters, vital signs, or ECG results were reported, and only 2 participants had potentially clinically significant hematology values at the end of the study or during the PK profiling periods.

Limitations of the study include the proportion of males, who constituted the majority of participants, although IBS‐D is more common in females.

## Conclusions

The results of this study suggest that eluxadoline has no clinically significant effect on CYP3A4 activity and is therefore unlikely to affect the PK of other CYP3A4 substrates. No dose adjustments are needed for CYP3A4 substrates when coadministered with eluxadoline.

## Conflicts of Interest

Financial arrangements of the authors with companies whose products may be related to the present report are listed below, as declared by the authors. R.B., D.M., E.K., and D.W. are employees of AbbVie Inc. and hold stock or stock options.

## Funding

Allergan plc, Dublin, Ireland (prior to acquisition by AbbVie Inc.) sponsored the study.

## Data Sharing

AbbVie is committed to responsible data sharing regarding the clinical trials we sponsor. This includes access to anonymized, individual, and trial‐level data (analysis data sets), as well as other information (eg, protocols and Clinical Study Reports), as long as the trials are not part of an ongoing or planned regulatory submission. This includes requests for clinical trial data for unlicensed products and indications.

This clinical trial data can be requested by any qualified researchers who engage in rigorous, independent scientific research, and will be provided following review and approval of a research proposal and Statistical Analysis Plan and execution of a Data Sharing Agreement. Data requests can be submitted at any time, and the data will be accessible for 12 months, with possible extensions considered. For more information on the process, or to submit a request, visit the following link: https://www.abbvie.com/our‐science/clinical‐trials/clinical‐trials‐data‐and‐information‐sharing/data‐and‐information‐sharing‐with‐qualified‐researchers.html.

## Supporting information



Supplemental Information
