# Supplementary material for: An Open‐Label Study to Evaluate the Effect of Eluxadoline on the Single‐Dose Pharmacokinetics of Midazolam in Healthy Participants
Source: Clin Pharmacol Drug Dev. 2022 Aug 8;11(11):1341–8. doi: 10.1002/cpdd.1150 (PMC9805131; doi:10.1002/cpdd.1150)
Supplement: Supplementary file 1 — Supplemental Information [file CPDD-11-1341-s001.docx]

**Supporting Information**

**Table S1.** Inclusion and Exclusion Criteria

| **Inclusion Criteria** | **Exclusion Criteria** |
| --- | --- |
| 1. Signed the informed consent form and had the mental capability to understand it 2. Healthy male or female, aged 18 to 45 years at the screening visit 3. If female, had a negative result from a serum pregnancy test at the screening visit and a negative result from a urine pregnancy test on day –1 4. If male, agreed to use an effective method of contraception and not have their partners become pregnant throughout the study, or had been sterilized for at least 1 year 5. If female (childbearing potential or non-childbearing potential), agreed to use an effective method of contraception and not become pregnant throughout the study. Females who were at least 2 years postmenopausal or who had had tubal ligation or hysterectomy were not considered to be of childbearing potential 6. A non-smoker (never smoked or have not smoked within the previous 2 years prior to the screening visit) 7. Body mass index ≥18 kg/m^2^ and ≤30 kg/m^2^ at the screening visit 8. Sitting pulse rate ≥50 bpm and ≤100 bpm during the vital sign assessment at the screening visit | 1. Known hypersensitivity to eluxadoline, opiates, or midazolam, or other benzodiazepines 2. Clinically significant disease state in any body system 3. Sitting systolic blood pressure ≥140 mmHg or ≤90 mmHg or sitting diastolic blood pressure ≥90 mmHg or ≤50 mmHg at the screening visit 4. Abnormal electrocardiogram results that were thought to be potentially clinically significant, or QT prolongation (QTcF ≥450 msec) at the screening visit 5. Positive test results for anti-HIV type 1 and type 2, hepatitis B surface antigen, or anti-hepatitis C virus at the screening visit 6. Abnormal and clinically significant results on physical examination, medical history, hematology, clinical chemistry, coagulation tests, or urinalysis 7. History of alcohol or other substance abuse within the previous 5 years. Positive test results for benzoylecgonine (cocaine), methadone, barbiturates, amphetamines, benzodiazepines, alcohol, cannabinoids, opiates, phencyclidine, or cotinine at the screening visit or day −1 8. Participation in any other clinical investigation using an experimental drug requiring repeated blood or plasma draws within 60 days prior to study treatment administration 9. Participation in a blood or plasma donation program within 60 or 30 days, respectively, prior to study treatment administration 10. Consumption of xanthine-containing compounds within 48 hours of study treatment administration 11. Consumption of alcohol within 72 hours before administration of study treatment 12. Consumption of beverages or food containing quinine, poppy seeds, vegetables from the mustard green family, or grapefruit, grapefruit juice, or Seville oranges within 14 days before administration of study treatment 13. Any clinical condition or previous surgery that might affect the absorption, distribution, biotransformation, or excretion of midazolam or eluxadoline 14. History of cholecystectomy 15. Employee or immediate relative of an employee, of the sponsor, any of its affiliates or partners, or the study center 16. Had taken any concomitant medications, including over-the-counter medications, within 14 days before study treatment administration, or hormonal drug products within 30 days before study treatment administration 17. Previously participated in an investigational study of eluxadoline 18. Breastfeeding 19. Unwilling to abstain from strenuous exercise from 48 hours prior to Day −1 until the end-of-study visit 20. Unable to understand the protocol requirements, instructions, and study-related restrictions, the nature, scope, and possible consequences of the clinical study 21. Unlikely to comply with the protocol requirements, instructions, and study-related restrictions |

**Measurements**

Blood samples were centrifuged at ≥2500 g for 10 minutes at approximately 4°C within 30 minutes from the time of the blood draw. After centrifugation, the plasma samples were harvested and flash-frozen in a dry ice and alcohol bath (with isopropyl alcohol) and stored at approximately –20°C.

Safety measures included adverse event (AE) recording, clinical laboratory determinations, vital sign parameters, electrocardiographic results, and physical examination findings. The investigator assessed the causal relationship of each AE to study treatment. AEs were coded using Medical Dictionary for Regulatory Activities version 19.1 and were reported from the time that the participant signed the informed consent form until 30 days after the last dose of study treatment. An AE that occurred during the treatment period was considered a treatment-emergent AE if it was not present before the first dose of study treatment or if it was present before the first dose of study treatment but increased in severity during the treatment period.

Eluxadoline quantitations in dipotassium ethylenediaminetetraacetic acid (K_2_EDTA) human plasma were determined using a validated liquid chromatography with tandem mass spectrometry (LC-MS/MS) methodology. The compounds of interest, eluxadoline (m/z 570.4 → 171.1) and its internal standard [^13^CD_3_]-JNJ-27018966 (m/z 574.4 → 171.1), were isolated from plasma using protein precipitation. A 50 µL extract aliquot was then combined with 100 µL of Nanopure water, and a homogenous aliquot (ranging from 5 to 20 µL) was then injected on an LC-MS/MS system. The high-performance liquid chromatography (HPLC) reversed phase chromatography utilized a stationary phase of Synergi Polar-RP (50 × 2 mm, 4 µm analytical column) and mobile phases A: methanol (MeOH)/water (H_2_O) [45/55, v/v] with 5 mM ammonium formate (HCOONH_4_), and B: MeOH/H_2_O (50/50, v/v) with 5 mM HCOONH_4_. Mobile phase A was pumped isocratically across the stationary phase with the HPLC eluant diverted to the mass spectrometer from 1.05 to 1.80 minutes with a 3.00-minute stop time. The mass spectrometer utilized a Turbo Ion Spray (electrospray ionization [ESI]+) source in positive mode. The assay range was validated from 0.100 to 100 ng/mL, which resulted in intra-assay quality control (QC) precision of coefficient of variation (%CV) of 1.66% to 4.61%, intra-assay QC accuracy relative error (%RE) of −4.70% to 3.60%, inter-assay QC precision of %CV of 1.62% to 8.59%, and inter-assay QC accuracy of %RE of −4.82% to 2.96%.

Midazolam and 1-hydroxy-midazolam quantitations in K_2_EDTA human plasma were determined using a validated LC-MS/MS methodology. The compounds of interest, midazolam (m/z 326.1 → 291.1) and its internal standard midazolam-d_4_ (m/z 330.1 → 295.1) plus 1-hydroxy-midazolam (m/z 342.1 → 203.1) and its internal standard 1‑hydroxy-midazolam-d_4_ (m/z 346.1 → 203.1), were isolated from plasma using protein precipitation. A 50 µL extract aliquot was then combined with 100 µL of 0.1% formic acid in Nanopure water, and a homogenous aliquot (ranging from 8 to 18 µL) was then injected on an LC-MS/MS system. The HPLC reversed phase chromatography utilized a stationary phase of ZORBAX Eclipse Plus C18 (50 × 3 mm, 3.5 µm analytical column) and mobile phases A: 5 mM HCOONH_4_ and 0.025% formic acid in water, and B: 0.1% formic acid in acetonitrile. A 4.5-minute gradient (0.6 to 2.4 mL/min variable flow rate) combined with a divert valve was run with the eluant pumped across the stationary phase and diverted to the mass spectrometer from 0.7 to 2.40 minutes. The mass spectrometer utilized a Turbo Ion Spray (ESI+) source in positive mode. The assay range was validated from 0.0500 to 25.0 ng/mL, which resulted in the following intra- and inter-day variability for midazolam: intra-assay QC precision of %CV of 1.09% to 6.47%, intra-assay QC accuracy of %RE of −1.59% to 4.45%, inter-assay QC precision of %CV of 2.34% to 5.10%, and inter-assay QC accuracy of %RE of −0.97% to 1.29%; and for 1-hydroxy-midazolam: intra-assay QC precision of %CV of 1.17% to 7.43%, intra-assay QC accuracy of %RE of −2.56% to 0.830%, inter-assay QC precision of %CV of 2.39% to 8.56%, and inter-assay QC accuracy of %RE of −1.06% to 2.19%.
